# GAMEPLAN4CARE: AN ONLINE TRANSLATION OF REACH II REFINED THROUGH EARLY STAKEHOLDER USABILITY TESTING

**DOI:** 10.1093/geroni/igz038.2493

**Published:** 2019-11-08

**Authors:** Jinmyoung Cho, Alan B Stevens, Thomas Birchfield, Sandhya Sanghi, Ashley Vernon, Marcia G Ory

**Affiliations:** 1 Baylor Scott & White Health, Temple, Texas, United States; 2 Texas A&M Center for Population Health and Aging, College Station, Texas, United States

## Abstract

Despite a number of evidences on the implementation of multicomponent education and support programs to improve the lives of dementia caregivers, there is still a need to adapt these interventions to various implementation settings and preferences of a new generation of caregivers. In this study, we present the development and usability testing of a dementia caregiver support program, GamePlan4Care (GP4C), an online translation of the Resources for Enhancing Alzheimer’s Caregiver Health II (REACH II) intervention. The goal of the GP4C is to create an online family caregiver support system that would facilitate delivery of an evidence-based skills-training and support for dementia caregivers with the potential of both scalability and sustainability. GP4C includes the full breadth of REACH II education and skill-building materials, delivered within an automated, online platform with integrated support from a Dementia Care Specialist via telephone/video conferencing. Dementia caregivers, community agency staff, and other experts are involved in usability testing to ensure acceptability of this new approach to intervention delivery. The software development is completed and usability testing is currently underway. The feasibility and success of this new modality of intervention delivery will be made possible by an innovative intervention design supported by appropriate technical and content elements. We will also present the strategies employed to adapt the intervention to an online platform capable of supporting caregiver self-directed exposure to therapeutic content, the results of usability testing with approximately 32 caregivers, and feedback from other external stakeholders on the feasibility of this approach.

